# The use of a new high-speed shielded curved drill is associated with improved intraoperative and clinical outcomes after cervical corpectomy and fusion procedures: a retrospective case series

**DOI:** 10.1186/s13018-023-03769-7

**Published:** 2023-05-17

**Authors:** John Peloza, Hani Malone, Erel Jacobian, Daniel E. Kolsky, Ran Harel, Richard D. Guyer, Michael A. Millgram, Ely Ashkenazi

**Affiliations:** 1 Center for Spine Care, 17980 Dallas Pkwy Ste 300, Dallas, TX 75287 USA; 2 Scripps Clinic Torrey Pines, 10666 N Torrey Pines Rd., La Jolla, CA 92037 USA; 3 Israel Spine Center, Assuta Medical Centers, Habarzel 20, 6971028 Tel Aviv, Israel; 4 Spine Surgery Unit, Department of Neurosurgery, Sheba Medical Center, 52662 Ramat-Gan, Israel; 5 Texas Back Institute, 6020 W Parker Rd Suite 200, Plano, TX 75093 USA

**Keywords:** ACCF, Anterior cervical corpectomy and fusion, Bone removal, Corpectomy, Fusion, Osteophyte

## Abstract

**Background:**

Anterior cervical corpectomy and fusion (ACCF) is an effective technique to address multi-level degenerative cervical myelopathy. However, as the number of surgical levels increases, the outcomes worsen with respect to complication rates, range of motion and length of surgery. This study aimed to determine the clinical outcome of ACCF procedures performed using a new distally curved and shielded drilling device.

**Methods:**

A retrospective study was conducted on 43 ACCF procedures in which the device was used for osteophyte removal. Patient files were reviewed to assess the early clinical results and complications following ACCF. Clinical outcomes were evaluated using patient neck and arm pain scores and SF-36 questionnaires. Hospitalization characteristics were compared with historical controls.

**Results:**

All procedures were uneventful and without major complications or neurological deterioration. Single-level ACCF procedures required an average of 71 min and followed by an average hospitalization of 3.3 days. Osteophyte removal, verified by intraoperative imaging, was satisfactory. Average neck pain score was improved by 0.9 points (*p* = 0.24). Average arm pain score was improved by 1.8 points (*p* = 0.06). SF-36 scores were improved in all domains.

**Conclusions:**

The new curved device enabled safe and efficient removal of osteophytes sparing adjacent vertebral removal in ACCF procedures, thus improving the clinical outcome.

**Supplementary Information:**

The online version contains supplementary material available at 10.1186/s13018-023-03769-7.

## Background

Degenerative cervical myelopathy (DCM) is a common degenerative disease of the intervertebral disks and vertebrae in the cervical spine. Patients with multi-level DCM are commonly treated by cervical corpectomy with good clinical results [1]. However, complications such as spinal cord or nerve roots damage, extensive blood loss, graft displacement or extrusion and others have been reported [2, 3]. For patients with multi-level DCM, corpectomy combined with discectomy (a hybrid decompression technique) provides an alternative option for nerve decompression and spinal reconstruction with lower complications rate as compared to anterior cervical corpectomy and fusion (ACCF).

Spinal osteophytes pressuring adjacent neural structures often cause neurological symptoms and pain and require surgical removal. These compressive osteophytes, located on the posterior aspect of the vertebral body, are difficult to access. Resection of posterior osteophytes has been reported to allow earlier decompression of the spinal cord and improved recovery. However, osteophyte removal is associated with increased incidence of injury to the spinal cord and the associated neurological risks [4, 5]. The surgical approach to cervical osteophyte remains controversial. The selection between the anterior, posterior and combined approaches is influenced by the location of the osteophyte, type of fracture or ligament injury and the overall alignment. ACCF is performed for patients with symptomatic, progressive cervical spinal stenosis and myelopathy. It is performed to remove the large, arthritic bone spurs that are compressing the spinal cord and spinal nerves. However, as the number of involved surgical levels increases, outcomes worsen with respect to blood loss, complication rates, decreased cervical range of motion (ROM) and length of surgery [6]. In addition, multi-level ACCF procedures are associated with increased mechanical failure rates and potential instability, longer procedure duration leading to soft-tissue retraction, increased pain and swelling, higher infection and reoperation rates and cost [7–11].

We previously described the hybrid decompression and fixation technique aiming to reduce the need for multi-level corpectomies [12]. This procedure involves a combination of corpectomy and discectomy in order to preserve an intact vertebra and motion segment within the area of the decompression, thus augmenting mechanical stability.

The Dreal® technology (Carevature Medical Ltd., Rehovot, Israel) is a powered, single-use platform to handle bony tissue during spinal surgeries. The device is inserted through the removed vertebral trough into the bony edge of the adjacent vertebral body osteophyte, anterior to the posterior longitudinal ligament. The device is used to drill into the osteophyte and not behind it, thus reducing the risk of spinal cord injuries and dural tears.

This study aimed to assess the safety and efficacy of the distally curved and shielded drilling device in patients suffering from cervical myelopathy treated with ACCF, including osteophytes removal with a minimum one-year follow-up. It was hypothesized that the use of this device will result in reduced complication rate in terms of range of motion, procedure duration, amount of blood loss, clinical parameters and the incidence of other complications.

## Methods

This retrospective study was approved by the institutional review board. Data of patients treated with ACCF with the aid of Dreal between 2013 and 2020 were collected via patient's records as well as by phone. Forty-three patients underwent ACCF and osteophyte removal using the device due to multi-level compression of the spinal cord and spinal nerves and were included. In order to estimate the potential benefit achieved due to the use of the device, similar historical cases were compared to the clinical case series. Since the device can potentially eliminate the need for an additional-level surgery, cervical procedures at three or four levels, with at least one corpectomy, were used as a control group. These procedures included either an additional corpectomy or additional discectomy to relieve the pressure at an adjacent level to the main corpectomy that can potentially be avoided due to the use of the device. All patients failed conservative therapies prior to surgical intervention.

The PROCESS (preferred reporting of case series in surgery) guidelines were observed in the preparation of this study [13].

### Surgical procedure

**Fig. 1:**
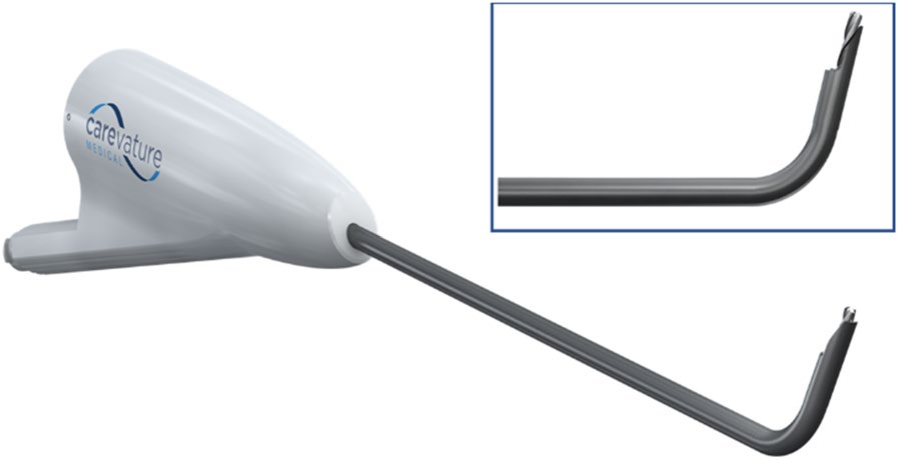
The Dreal® model used for the procedure. Top right: enlarged view of the distal tip of the device

Under general anesthesia, polymodal somatosensory and motor neuromonitoring and electromyography root monitoring, the patients are placed in the supine position with neck extension. A standard left-sided anterior cervical approach and one-level cervical corpectomy are performed. Following the corpectomy, the osteophytes are removed using the device, a distally curved high-speed drilling device (Dreal®, Carevature Medical Ltd., Rehovot, Israel), presented in Fig. 1. The device is FDA-cleared for bone removal in spinal surgery, has been shown to be associated with a reduced dural tear rate, compared with other bone removal devices, and can be used for multiple spinal indications [14, 15].

**Fig. 2:**
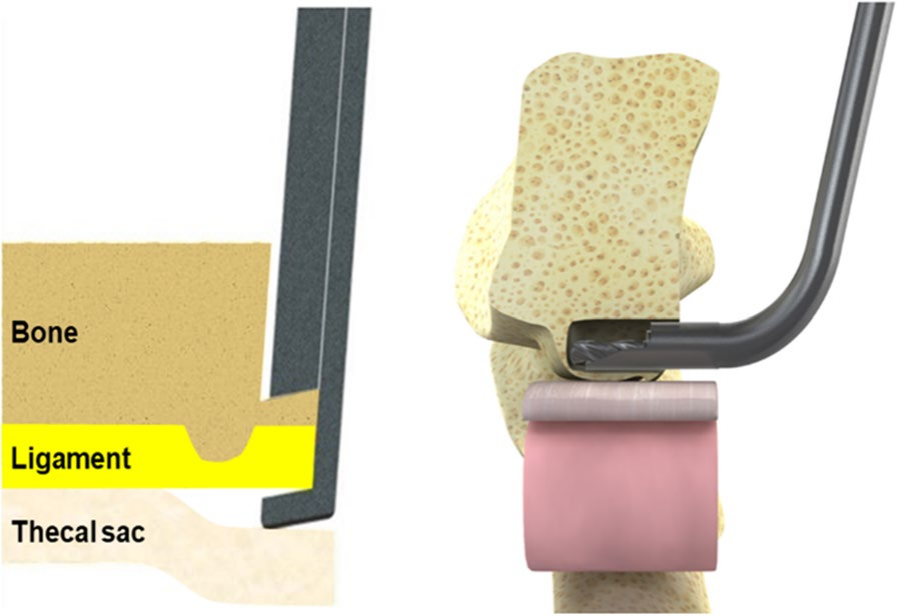
Osteophyte drilling approach. Left: Traditional tools, the osteophyte is captured from behind, with a risk to neural structures. Right: New device, the device is used to drill into the osteophyte, while the neural structures are protected by the shield and potentially by the posterior longitudinal ligament

The device is inserted through the removed vertebral trough into the bony edge of the adjacent vertebral body osteophyte, anterior to the posterior longitudinal ligament. As opposed to the osteophyte removal using traditional tools, such as Kerrison rongeurs, the device is not placed between the osteophyte and the dura or inserted into the central canal. Instead, the device is used to drill into the osteophyte and not behind it, as shown in Fig. 2, thus reducing the risk of spinal cord injuries and dural tears. The device holds two advantages over the use of Kerrison rongeurs for this purpose. First, its curved shape and length at the distal aspect allow deeper and more precise decompression without excessive removal of supporting bone structures. Second, the drilling edge is shielded on one side in order to protect the spinal cord.

**Fig. 3:**
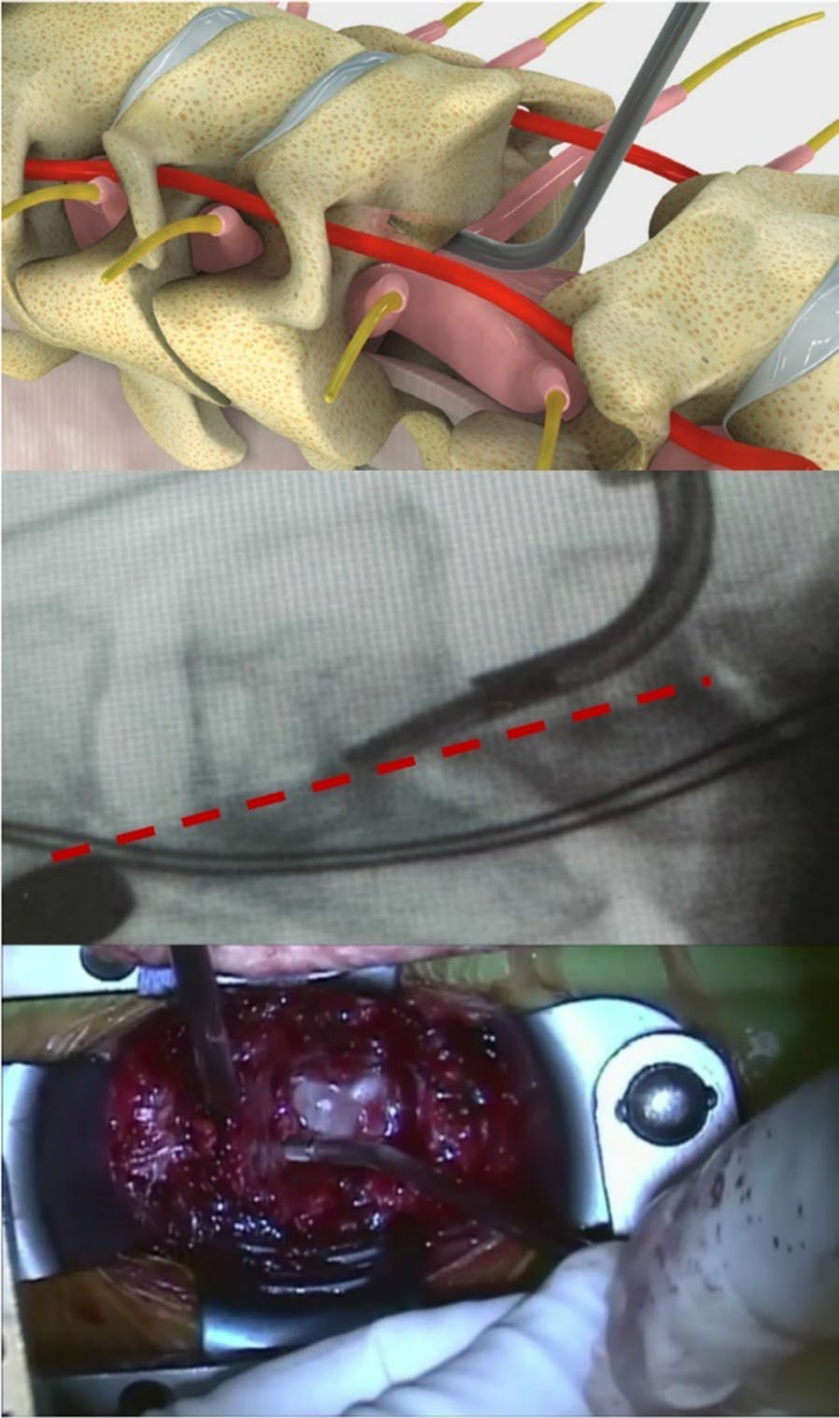
Device approach and orientation during osteophyte removal. Top: rendering to illustrate access to the osteophyte via the one-level corpectomy channel. Middle: O-arm image of the 2-mm model—the posterior edge of the vertebra is marked by a red dashed line. Bottom: view from the surgeon’s perspective. In all images, the cranial side is to the left and the drilling tip of the device is inside the osteophyte at the posterior side of the vertebral body

The device is placed anterior to the thecal sac and posterior longitudinal ligament and used to drill into the osteophytes parallel to the thecal sac (Fig. 3). The surgeon continues to drill forward into the osteophyte, moving in a superior–inferior direction until the osteophyte becomes loose and detached from the vertebral body and sufficient decompression is achieved. Next, if the osteophyte and the remaining ossified posterior longitudinal ligament (OPLL) are not attached to the dura, they are removed. If there is adhesion of an osteophyte bone fragment or OPLL to the dura, it is left to dissolve attached to the dura and isolated from remaining bone tissue. Since deflection of the underlying thecal sac is not required, the risk of a compressive injury is reduced. Osteophyte removal may be verified using imaging or nerve probes. Discectomies above and below the resected vertebra are performed and implant insertion completes the procedure. As with any procedure performed in close proximity to neural structures, the surgeon should be familiar with the relevant anatomy and work according to its limitations. The device has an integrated irrigation port, allowing a constant flow to avoid excessive heating.

The diameter of the device’s tip was 2 mm in most of the procedures conducted (*n* = 36). An older model with a 3-mm diameter was used during the first seven procedures. The surgeons obtained initial experience with the device on simulating models (Sawbones, Pacific Research Company, Vashon Island, WA, USA) and cadaver workshops.

The described technique is demonstrated in an additional movie file taken during a live procedure [Additional file 1: Video 1]. The device model used for the video was the initial version, which included a 3-mm-diameter tip, larger than the 2-mm-tip model that is currently used.

All procedures were conducted by five trained and experienced spine surgeons in a single center. The decision on whether to use the device in a procedure was usually made based on preoperative imaging, when a large osteophyte was observed at the posterior side of the vertebra. In some procedures, the decision to use the device was made intraoperatively, if the surgeons noticed that sufficient decompression could not be achieved safely using standard tools and methods.

The procedures were assessed using intraoperative parameters, such as operation time, blood loss and postoperative parameters, including complication rates and symptom relief. The clinical outcomes were assessed preoperatively and at follow-up using visual analogue scale (VAS) and SF-36 questionnaires. SF-36 physical and mental component scores (PCS and MCS, respectively) were calculated using the United States factors [16]. Unfortunately, due to the historical nature of most of the control procedures, clinical outcome questionnaires were only available for the device group.

Statistical analysis was conducted using a statistical software (JASP, JASP Team, 2019). The paired samples Student’s *t*-test was used to compare preoperative and postoperative pain and disability scores. All tests were two-tailed. The potential effects of patient age and sex on the outcome scores were evaluated using a one-way ANCOVA (analysis of covariance) test. A cutoff P value of 0.05 was set to determine statistical significance.

## Results

Forty-three patients underwent ACCF procedures that included osteophyte removal using the device. Ten procedures were conducted without the device and included an additional surgical level. Patient characteristics are presented in Table 1. As shown in Table 1, the main indication of the patients was myelopathy with or without radiculopathy. Several patients also presented with dominant radiculopathy symptoms. Yet, these patients suffered from additional myelopathic symptoms as well and were therefore included in this study. Intraoperative parameters are presented in Table 2. The mean age was 58 (range 40–84) years in the device group and 59 (range 29–83) years in the control group (*p* = 0.8). Single-level ACCF in the device group was usually performed without additional corpectomies or discectomies. The most commonly resected level was C5 or C6. Surgery time was available for 37 patients (95%) in the device group and for all patients in the control group.

**Table 1:** Patient characteristics

	Dreal	Control
Age	58 years (range 40–84)	59 years (range 29–83)
*Sex*		
Female	16 (37%)	5 (50%)
Male	27 (63%)	5 (50%)
*Smoking status*		
Yes	14 (33%)	2 (20%)
No	29 (67%)	8 (80%)
*Procedure type*		
ACCF	32 (74%)	
ACCF + ACDF at adjacent level	10 (23%)	8 (80%)
ACCF, skip level	1 (2%)	
Two-level ACCF		2 (20%)
*Resected level*		
C4	10 (23%)	1 (10%)
C5	13 (30%)	4 (40%)
C6	19 (44%)	3 (30%)
C4 + C6	1 (2%)	
C5 + C6		2 (20%)
*Indication*		
Cervical myelopathy	34 (79%)	9 (90%)
Cervical myelopathy and radiculopathy	9 (21%)	1 (10%)
*Comorbidities*		
Diabetes	5 (12%)	1 (10%)
Cardiac disease	7 (16%)	1 (10%)
Coagulation disorders	1 (2%)	
Chronic obstructive pulmonary disease (COPD)	5 (12%)	3 (30%)
Hypertension	14 (33%)	3 (30%)
Obesity	1 (2%)	
Osteoporosis		2 (20%)

**Table 2:** Intraoperative and postoperative parameters

	Dreal	Control
*Surgery time (skin to skin)*		
ACCF	71 min (sd = 12, n = 29)	
ACCF + ACDF at an adjacent level	91 min (sd = 15.6, n = 9)	94 min (sd = 31, n = 8)
ACCF, skip level	131 min (n = 1)	
Two-level ACCF		80 min (sd = 13, n = 2)
*Blood loss*		
0-300 ml	n = 42 (98%)	n = 10 (100%)
400 ml	n = 1 (2%, the ACCF skip-level procedure)	
*Hospitalization*		
ACCF	3.3 days (sd = 1.1, n = 32)	
ACCF + ACDF at an adjacent level	3.9 days (sd = 1.7, n = 10)	3.5 (sd = 0.5, n = 8)
ACCF, skip level	3 days (n = 1)	
2ACCF		5.5 days (n = 2)

Bone removal was satisfactory in all cases, as assessed by the intraoperative imaging. Patients were required to use neck braces during driving only, a substantially reduced requirement compared with two-level corpectomy procedures. All procedures were uneventful and without Dural tears. Two revision surgeries occurred in the device group (4.7%): One patient suffered from an epidural infection and was readmitted and operated again to drain the epidural abscess and for an additional-level anterior cervical discectomy and fusion (ACDF). The second patient was readmitted due to postoperative arm pain and weakness and was reoperated for an additional foraminotomy at the corpectomy level (using another model of the Dreal). Both reoperations were not related to the use of the device.

Comparing single-level ACCF procedures conducted with the device to control three-level procedures (ACCF with adjacent-level ACDF or two-level ACCF procedures) reveals that saving an additional surgical level results in a statistically significant reduction of 20.4 min in surgery duration (*p* < 0.01) and a potential reduction in hospitalization duration of 0.65 days (*p* = 0.17).

Table 3 shows the changes in the clinical outcome parameters between the preoperative questionnaires and the latest-follow-up questionnaires (average time from surgery: 628 days, range: 141–1268 days) for the device group. Changes were calculated for all patients with both preoperative and postoperative scores available.

**Table 3:** Patient outcome metrics, device group: average values and standard deviations

		*N*	Preoperative		Postoperative		Difference		*p* value
VAS	Neck pain	20	5.6	(3.0)	4.7	(3.2)	− 0.9	(3.4)	0.23
	Arm pain	20	6.2	(2.7)	4.5	(3.8)	− 1.8	(4.0)	0.06
SF-36	Physical functioning	21	52.1	(25.0)	63.6	(26.9)	+ 11.5	(28.8)	0.08
	Role limitations due to physical health	19	26.3	(36.8)	36.8	(44.4)	+ 10.5	(50.9)	0.38
	Role limitations due to emotional problems	19	40.4	(46.6)	42.1	(50.7)	+ 1.8	(59.3)	0.9
	Energy/fatigue	20	40.6	(18.3)	45.3	(28.0)	+ 4.7	(26.8)	0.45
	Emotional well-being	20	51.6	(21.4)	69.1	(28.7)	+ 17.5	(26.0)	0.01
	Social functioning	20	49.4	(32.1)	58.8	(34.7)	+ 9.4	(42.9)	0.34
	Pain	20	36.8	(23.6)	49.9	(32.8)	+ 13.1	(38.5)	0.14
	General health	21	59.0	(22.1)	62.4	(27.2)	+ 3.3	(25.3)	0.55
	Health change	21	26.2	(24.3)	76.2	(27.9)	+ 50.0	(37.1)	< 0.01
	Physical component score (PCS)	17	38.5	(18.3)	47.6	(25.8)	+ 9.1	(27.7)	0.19
	Mental component score (MCS)	17	49.9	(19.9)	60.1	(22.3)	+ 10.1	(22.8)	0.08

As DCM is a progressive condition, the main goal of the surgery is to halt its progression and not to substantially improve the pain and disability [17]. However, despite the relatively long follow-up period, some improvement was noted in the clinical outcome parameters as well: The average patient neck and arm VAS scores were both slightly improved by 0.9 and 1.8 points, respectively (*p* = 0.23 and *p* = 0.06). Patient disability was improved as indicated by the SF-36 scores, which were improved in all domains. PCS and MCS scores were improved by 9.1 and 10.1 points, respectively. The most prominent changes were noted in the emotional well-being (+ 17.5, *p* = 0.01) and health change (+ 50.0, *p* < 0.01) domains.

A one-way ANOCVA test was conducted to study the effect of patient age and sex on the improvement of the pain VAS scores. The analysis revealed a statistically significant effect of patient sex on the neck pain improvement (*p* = 0.03, with a post hoc difference of 3.4 points). Women who completed both questionnaires were younger (50 years old compared with 63 years old, *p* < 0.01) and had a higher single-level procedures rate (87.5% compared with 66.7%, *p* = 0.29), which could explain this difference.

In addition, based on examination of medical records, the neck or arm pain increase experienced by some of the patients was caused by development of stenosis on different levels or areas. As shown in Table 3, the variability in preoperative pain and disability scores is high and may be a result of the grouping of various indications (patients with pain, compared with patients without pain but with a disability, and patients operated upon due to recent trauma).

**Fig. 4:**
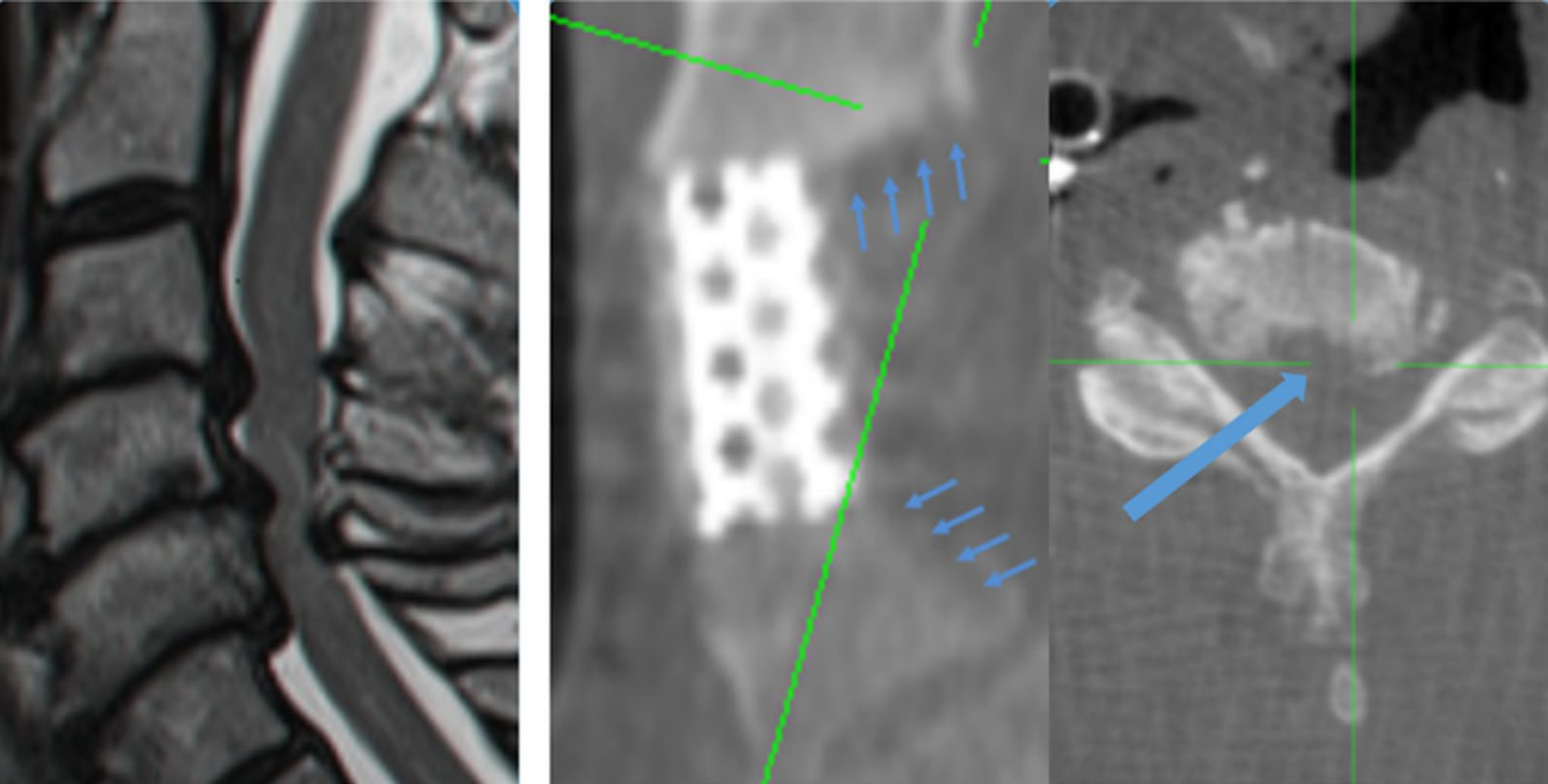
Imaging scans of a patient treated for C3-C6 stenosis. Left: preoperative scan. Right: intraoperative scans, showing the implant and the areas treated using the device, the adjacent vertebrae remained intact except for the removed osteophytes (marked by arrows). The transverse view image shows the treated region of the C5 vertebra, marked by the square in the sagittal view image

Figure 4 shows the imaging scans of a patient suffering from severe stenosis at levels C3–C6. The patient was otherwise a candidate for a C4–C5 ACCF procedure, as shown in the preoperative MRI scan (left). Yet in the procedure using the device only C4 was resected, and the posterior osteophytes at C5–6 were removed according to the described protocol, therefore avoiding a C5 corpectomy. The intraoperative scan (right) demonstrated that the osteophytes were successfully removed.

**Fig. 5:**
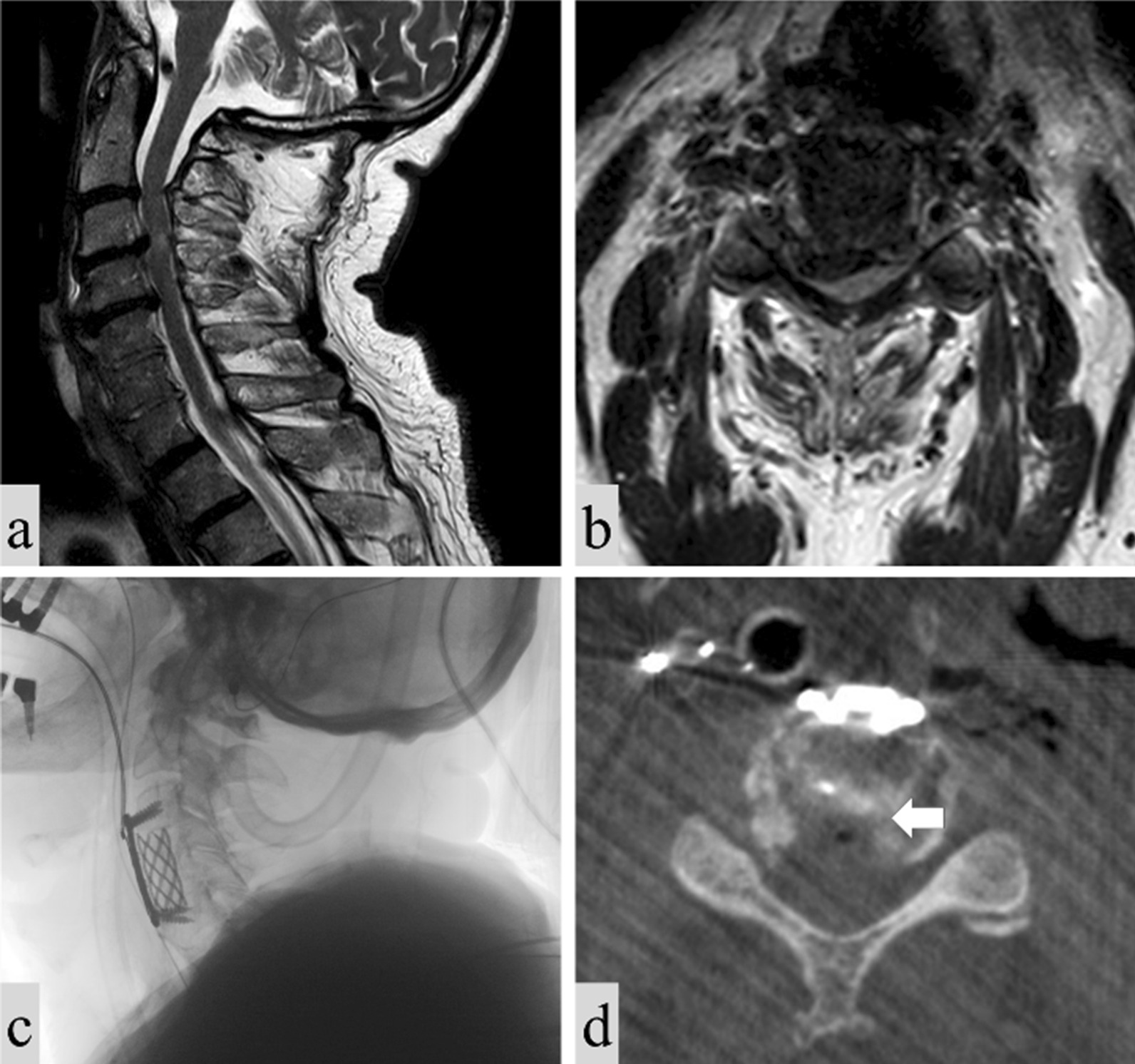
Imaging scans of a patient treated for C3–C5 stenosis. **a** Preoperative MRI, sagittal view; **b** preoperative MRI, transverse view; **c** intraoperative x-ray, sagittal view; **d** intraoperative CT scan, axial view of the treated region, demonstrating successful removal of the osteophyte (marked by an arrow) without bone removal at the vertebral posterior side or compromising the integrity of the adjacent vertebrae

Figure 5 shows the preoperative and intraoperative scans of an 84-year-old male patient who underwent the described procedure. The patient suffered from multiple injuries following a car accident 3 months prior to the surgery. The patient presented with bilateral arm pain, weakness and paraesthesias in four limbs with unstable gait. The preoperative MRI scan showed cervical cord compression at C3–4 with calcified disk and myelomalacia, C4–5 disk herniation with mild cord compression and spontaneous C5–7 fusion. The patient underwent a C4 corpectomy with discectomies at C3–4 and C4–5 and osteophyte removal at C3 according to the described protocol. Successful removal of the osteophyte from the adjacent vertebra was achieved without excessive bone removal, avoiding resection of two vertebral bodies. At the postoperative examination, arm weakness, the paraesthesias and the gait were all improved.

**Fig. 6:**
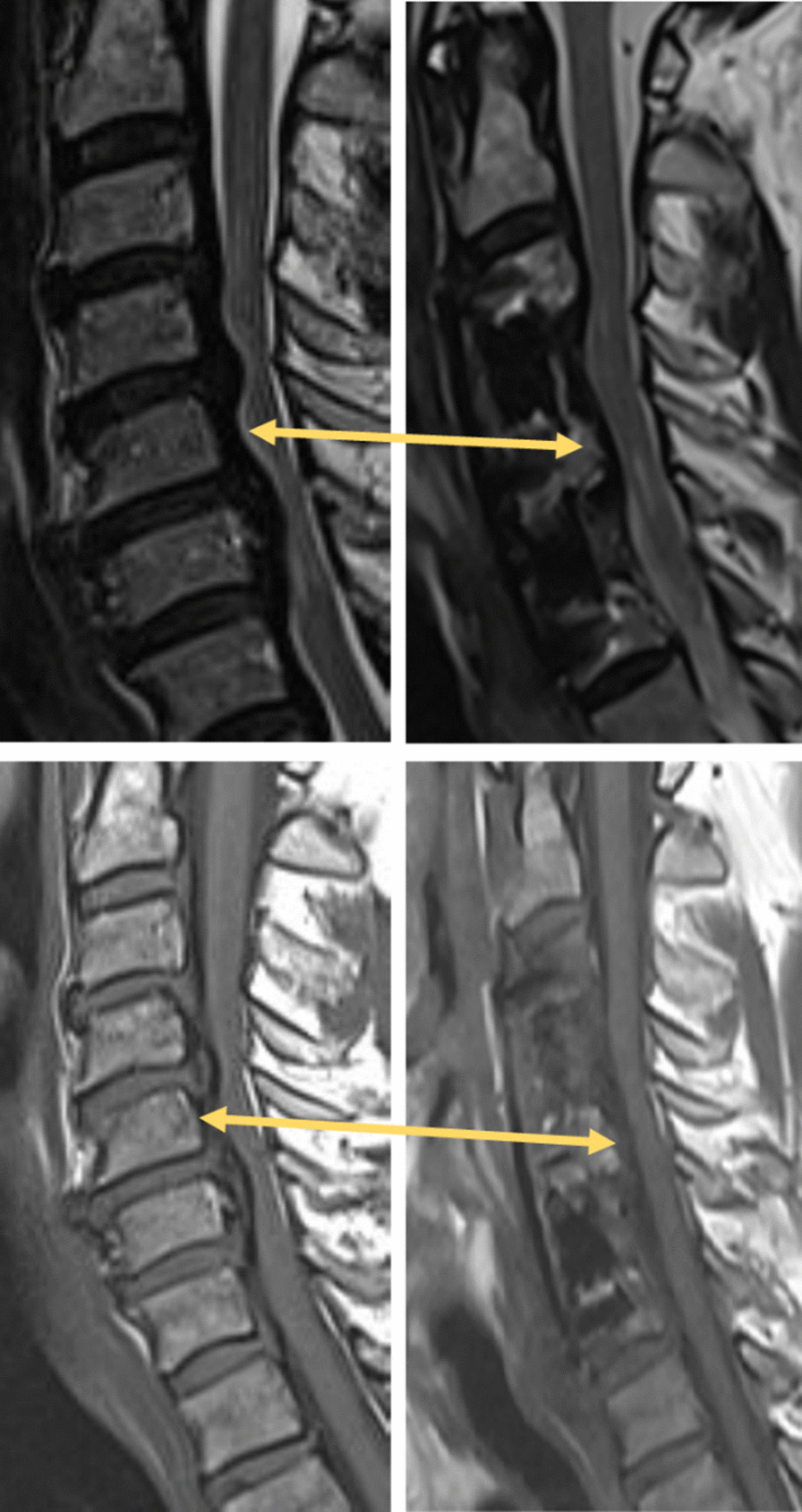
MRI scans of a patient treated for C4-C7 stenosis. Top: T2. Bottom: T1. Left: preoperative scan. Right: postoperative scans, showing the implant and the areas treated using the device. The arrows mark the area posterior to the preserved vertebra demonstrating the removal of cord compressive tissue by the Dreal. Note the diameter of the cord in the T1 images pre- and post-surgery

Figure 6 shows the preoperative and intraoperative scans of a 59-year-old male patient who underwent skip-level corpectomy. The patient suffered from motor weakness for eight years and was recommended for surgery by another surgeon three years prior to his visit to the clinic but declined. The patient presented with weakness in the right arm and leg. His gait was spastic, and he required a walking cane to ambulate. The right arm was almost completely paralyzed with significant muscular atrophy in the deltoid muscle, as well as in the triceps and biceps. Examination also revealed hyperreflexia and pyramidal Babinski, Hoffman and Tremner signs. The preoperative MRI scan showed severe stenosis and cord compression from C4 to C6 with high-intensity signal in the spinal cord. The patient underwent a C4 and C6 corpectomy with C3–4, C4–5, C5–6 and C6–7 discectomies and osteophyte removals according to the described protocol. Two weeks after the procedure the patient demonstrated improved gait and right arm motor power. At 6-month follow-up, the gait and arm strength were improved. The patient complained of neck stiffness. A subsequent MRI revealed that cord compression was released, as demonstrated in Fig. 6. Due to continuation of some of the complaints, the patient was referred to a pain management clinic and physiotherapy, without further complaints.

## Discussion

The principal results of this study demonstrated that all procedures were uneventful and without major complications or neurological deterioration. Single-level ACCF procedures required an average of 71 min and followed by an average hospitalization of 3.3 days. Osteophyte removal, verified by intraoperative imaging, was satisfactory. Average neck pain score was improved by 0.9 points. Average arm pain score was improved by 1.8 points. SF-36 scores were improved in all domains.

Cervical stenosis can be surgically addressed through different approaches. Osteophyte removal at the posterior aspect of the vertebral body may lead to increased morbidity due to the proximity to neural structures. Among anterior approaches, ACCF offers improved decompression ability and fusion rates but is associated with a higher complication rate, compared with ACDF [5, 18, 19]. Multiple studies explored the potential benefits and complications of this procedure. Lin et al. [3] reported a procedure length of 125 min and a complication rate of 22.4%, similar to the rates reported by Liu et al. in their study (22.4% complication rate and 123 min) [20]. Oh et al. [21] reported an average operation length of 210 min, hospitalization duration of 16.82 days, a complication rate of 18% and a reduction in neck and arm pain scores of 0.36 and 3 points, respectively.

The average surgery duration in the current study was shorter than reported surgical durations in published meta-analyses, ranging between 116 and 268 min [22, 23]. The operation time of two-level ACCF procedures has been reported at 116 min and longer [24, 25]. Avoidance of a multi-level ACCF procedure could potentially be accomplished using the described technique, as it allows the surgeon to access and remove the osteophyte in the vertebra adjacent to the resected vertebra, without having to remove it as well.

Substantial pain score improvements were described by Yang et al. [26], who reported a reduction of 4–5.8 points in the pain score, depending on follow-up time and the type of cage used. Hwang et al. [27] reported a pain score decrease of 5.7 with a length of stay of 15.6 days. The average pain score reduction in this study was somewhat lower than some of the literature findings. This difference could result from the lower average age of the patients operated in these studies (47 and 54 years), compared with the current study (57.9 years for the patients who completed both questionnaires). Other differences in patient characteristics, such as sex, percentage of smokers and duration of symptoms, may also affect the clinical outcome.

Two complications and revision surgeries were recorded in this study (4.7%), while the reported complication rates of ACCF procedures are approximately 20%. It is therefore possible that the use of the new safer device contributed to this improvement. Comparison to a control group, operated upon by the same surgeons using traditional tools and methods, and increasing the sample size could allow for a more reliable comparison. However, such a comparison could not be performed by the authors, as they have been using the device for the described procedures since 2013, as also evident by the limited number of available control procedures in this institution. Therefore, any retrospective comparative study would be affected by other factors which changed during this time period. In addition, some of the authors of this study are affiliated with the company manufacturing the device, creating an inherent bias to the study. These authors were among the early users of the device, which allowed them to accumulate this relatively large case series considering the uncommon procedure type. Based on the experience of the authors, the use of the device improves the safety and efficacy of the procedure. For this reason, conducting a prospective, randomized, controlled study would be considered inappropriate by the authors as they believe it would negatively affect the outcome of the control group. Such a study would be very valuable if conducted by unbiased surgeons in a different institution.

This study has several limitations inherent to its retrospective uncontrolled design. First, patient's number is relatively small. Study population is heterogeneous with different symptoms, physical and imaging findings, concomitant pathologies and surgical indications. The follow-up period is short and did not extend through full recovery in some patients. These limitations cause difficulty in drawing conclusions or calculating statistical tests regarding the functional outcomes. Nevertheless, because of the novelty of the device and technique, it is important to report even these limited findings.

## Conclusions

The described device offers a new technique for treatment of multi-level degenerative spondylotic myelopathy, allowing safe osteophyte removal while reducing the need for additional corpectomy(ies) in most of the procedures conducted. Less postoperative neck and arm pain was reported in patients that underwent ACCF using The Dreal® technology. Based on the current study results, the use of a curved and shielded drilling device is safe and effective and offers the potential to significantly improve the clinical outcome by shortening the procedure and reducing surgical morbidity. The device offers a safer approach to osteophyte removal due to its distally curved design, its shield and its capability to resect dorsal bony pathology through a drilling technique that is anterior to the posterior longitudinal ligament. In combination, these features serve to protect adjacent neural tissues and the dura. In addition, the narrow width of the device tip affords the possibility of a safer surgical operation.

The technique presented enables safe and efficient removal of osteophytes while eliminating the need to resect adjacent vertebral body(ies), thus maximizing patient range of motion.

## Supplementary Information

**Additional file 1**.** Video 1**. Device use during a procedure: animation and surgical video.

## Data Availability

The datasets generated and analyzed during the current study are not publicly available due to patient privacy.

## Abbreviations

ACCF: Anterior cervical corpectomy and fusion
ACDF: Anterior cervical discectomy and fusion
ANCOVA: Analysis of covariance
COPD: Chronic obstructive pulmonary disease
DCM: Degenerative cervical myelopathy
FDA: Food and Drug Administration
MRI: Magnetic resonance imaging
OPLL: Ossified posterior longitudinal ligament
PROCESS: Preferred reporting of case series in surgery
ROM: Range of motion
VAS: Visual analogue scale
